# Beclin-1 knockdown decreases proliferation, invasion and migration of Ewing sarcoma SK-ES-1 cells via inhibition of MMP-9

**DOI:** 10.3892/ol.2020.12372

**Published:** 2020-12-11

**Authors:** Conglin Ye, Xiaolong Yu, Xuqiang Liu, Ping Zhan, Tao Nie, Runsheng Guo, Hucheng Liu, Min Dai, Bin Zhang

Oncol Lett 15: 3221-3225, 2018; DOI: 10.3892/ol.2017.7667

The authors drew to our attention that they had noticed that Fig. 2 in the above published article contained an error: Essentially, the same data panel had been selected for the 0 h/Con and 0 h/si-con experiments in Fig. 2C on p. 3224. Furthermore, an interested reader drew to our attention that a pairing of control β-actin bands used in Figs. 1 and 3 may have been derived from the same original source.

After having re-examined their data, the authors were able to identify the correct images for the 0 h/Con and 0 h/si-con experiments, and the corrected version of Fig. 2, including the correct data for Fig. 2C, is shown opposite. Furthermore, alternative data are also shown for Figs. 1 and 3, as presented below and opposite. Note that the alterations made to these Figures do not affect the results or the conclusions reported in this paper, and all the authors agree to this Corrigendum. The authors thank the Editor for presenting them with the opportunity to publish this Corrigendum, and apologize to the Editor and to the readership of the Journal for any inconvenience caused.

## Figures and Tables

**Figure 1.**
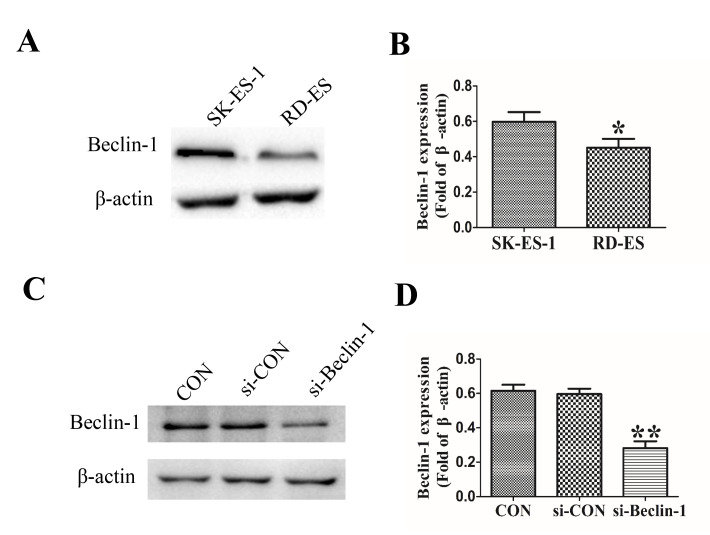
Endogenous expression of Beclin-1 in the SK-ES-1 and RD-ES cell lines was evaluated using western blot analysis. (A) Expression of Beclin-1 was markedly increased in the SK-ES-1 cell line compared with the RD-ES cell line. (B) Quantification of the western blotting confirmed that the expression of Beclin-1 was significantly decreased in RD-ES cells compared with SK-ES-1 cells. (C) Protein levels of Beclin-1 were determined using western blotting once SK-ES-1 cells were transfected with si-beclin-1 or si-con vectors for 48 h. (D) Beclin-1 expression was significantly decreased in the si-beclin-1 group compared with the blank control group. *P<0.05, **P<0.01 compared with control group. Con, blank control group; si-con, SK-ES-1 cells transfected with blank plasmid; si-beclin-1, SK-ES-1 cells with Beclin-1 knocked down.

**Figure 2.**
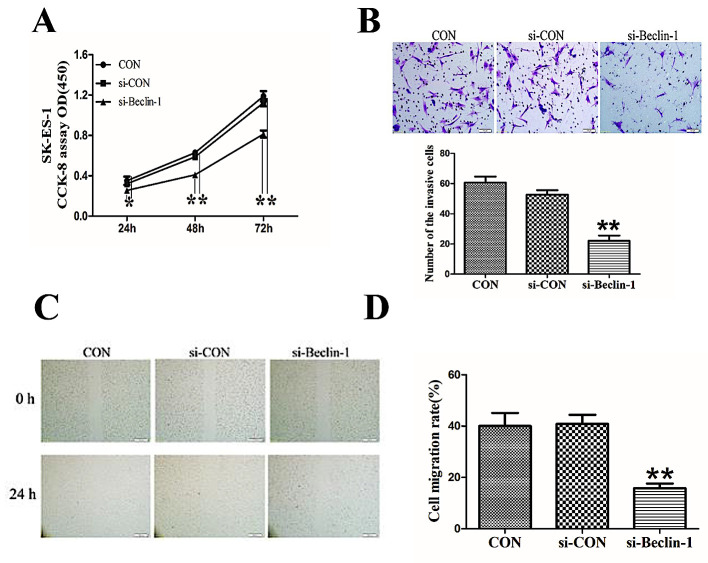
Knockdown of Beclin-1 suppressed proliferation, invasion and migration of SK-ES-1 cells. (A) CCK-8 assay was performed to examine SK-ES-1 cell proliferation. Knockdown of Beclin-1 significantly inhibited SK-ES-1 cell proliferation. (B) A Matrigel-coated Transwell assay was conducted to confirm the invasion of SK-ES-1 cells (magnification, ×100). Knockdown of Beclin-1 significantly repressed the invasion of SK-ES-1 cells. (C) A wound healing assay was conducted to determine the migration of SK-ES-1 cells (magnification, ×40). (D) Knockdown of Beclin-1 significantly repressed the migration of SK-ES-1 cells. **P<0.01 compared with the control group. CCK-8, Cell Counting Kit-8; con, blank control group; si-con, SK-ES-1 cells transfected with blank plasmid; si-beclin-1, SK-ES-1 cells with beclin-1 knocked down.

**Figure 3.**
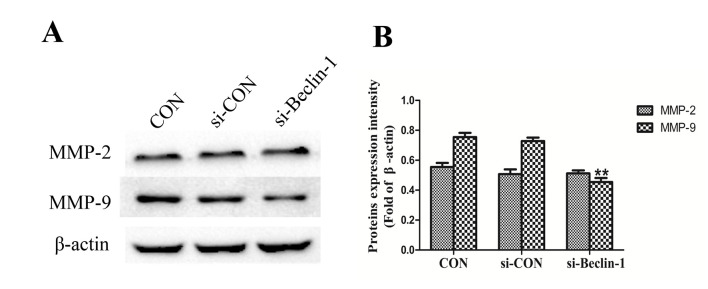
Beclin-1 knockdown decreases MMP-9 expression. (A) Western blot analysis was performed to investigate the effect of Beclin-1 knockdown on the expression of MMP-2 and MMP-9, since it is generally acknowledged that they are associated with tumor invasion and metastasis. (B) MMP-9 expression was significantly decreased in the si-beclin-1 group compared with the Con group, although no significant difference in the expression of MMP-2 was observed. **P<0.01 compared with the control group. MMP, matrix metalloproteinase; con, blank control group; si-con, SK-ES-1 cells transfected with blank plasmid; si-beclin-1, SK-ES-1 cells with beclin-1 knocked down.

